# P29 Understanding penicillin allergy assessment and delabelling massive open online community

**DOI:** 10.1093/jacamr/dlae136.033

**Published:** 2024-08-23

**Authors:** Neil Powell, Alicia Quinn, Chris Weaving, Jonathan Sandoe, educational team, Rashmeet Bhogal, Shuayb Elkhalifa, Kimberly Blumenthal, Jacqueline Sneddon, Sarah Tonkin-Crine, Jason Trubiano, Cosby Stone, Joanna Stollings

**Affiliations:** BSAC, Birmingham, UK; BSAC, Birmingham, UK; BSAC, Birmingham, UK; BSAC, Birmingham, UK

## Abstract

**Background:**

PenA records are associated with antibiotic prescribing that is potentially less effective and more harmful. The paucity of allergy specialists worldwide has prompted non-allergist delivered penicillin allergy delabelling (PADL). To facilitate non-allergist delivery of PADL, a massive open online course (MOOC) was developed with objectives to raise awareness and provide healthcare workers with the requisite knowledge and skills to deliver PADL.

**Methods:**

Developmental support and funding for the MOOC was provided by BSAC. An educator panel was convened with topic experts from the USA, UK and Australia representing infectious diseases, allergy, pharmacy, medical microbiology and health psychology. Key learning objectives were defined, the course structure was agreed, and content was developed and peer reviewed by panel members and BSAC. The MOOC was launched and hosted on FutureLearn. Data on learner numbers, demographics and feedback were collected to measure course uptake and effectiveness in meeting the objectives.

**Results:**

The 2 week course entitled ‘Understanding Penicillin Allergy Assessment and Delabelling’ with 6 h of learning was published 20 November 2023 on FutureLearn. The course was accredited by the Royal College of Pathologists for 6 CPD points. As of 10 April 2024, there were 1599 learners from 119 countries, which were: 16% medical doctors, 2% surgical doctors, 3% other doctors, 18% pharmacists, 11% nurses, 34% students, 1% physicians associate (PA), 1% nurses associate and 15% other. The top five countries by joiner count are UK (466), India (118), Pakistan (60), Italy (44) and Egypt (44). Of those that completed the course and responded to the end-of-course survey, 40/65 (62%) rated the course better than expected, 24 (37%) had their expectations met and 1 (1.5%) rated the course worse than expected. Overall, 61 (94%) gained new knowledge or skills, 29 (45%) applied their learnings and 49 (75%) reported sharing their learnings with others. Examples of learner comments:

“*Truly, the best penicillin allergy explanation I have ever come across. So detailed and well illustrated. Thank you!*”

“*Fantastic course! I’m going to recommend every health care professional at my organisation complete this as part of my Antimicrobial Stewardship training. Thank you for putting it together.*”

**Conclusions:**

The MOOC has reached a broad range of learners from a variety of disciplines in a range of countries globally. The reception has been very positive, with comments stating that knowledge of penicillin allergies had improved and applied to improve local PADL services—a direct indication of meeting the learning outcomes.

